# Determination of Tributyltin in the Marine Environment

**DOI:** 10.6028/jres.093.043

**Published:** 1988-06-01

**Authors:** R. G. Huggett, M. A. Unger, F. A. Espourteille, C. D. Rice

**Affiliations:** Virginia Institute of Marine Science, School of Marine Science, College of William and Mary, Gloucester Pt., VA 23062

## Introduction

Tributyltin (TBT) is a biocide used in antifouling paints to protect hulls from nuisance organisms such as barnacles, worms and algae. The use of TBT paints has increased over the past decade due to its effectiveness as an antifoulant which is related to its toxicity. Water concentrations of less than 100 ng L^−1^ have been shown to harm some aquatic species in laboratory tests and observations in the natural environment indicate that levels below 10 ng L^−1^ may be harmful. Tributyltin is bio-concentrated by many species to levels of one thousand, or more, times ambient water concentrations. Sediment-water partitioning coefficients for TBT of 100–10,000 have been reported [1]. The extreme toxicity of TBT challenges the analytical chemist to accurately and precisely determine ambient TBT concentrations in water at or below 1 ng L^−1^ and in sediments and tissue at concentrations ranging from µg kg^−1^ to mg kg^−1^.

## Methods and Procedures

Tributyltin is often derivatized to convert the substance to a more volatile compound to facilitate analyses [2,3]. Our methodology for water involves liquid-liquid extraction of an acidified sample with tropolone and n-hexane. This is followed by the generation of n-hexyltributyltin, using the Grignard reagent, n-hexyl magnesium bromide. The derivatized extract is then cleaned by fluorisil column chromatography and analyzed by capillary gas chromatography with flame photometric detection or gas chromatography-mass spectrometry. Tripentyltin chloride is added as an internal standard before extraction of the sample [4].

The methodology for quantification of TBT in sediments and tissue differs from the water procedure only in the extraction steps. Anhydrous sodium sulfate and precipitated silica are added to wet tissue or sediment to dessicate the sample. It is then frozen to lyse cells and ground to a fine powder consistency. The TBT is removed from the sample by soxhlet extraction with n-hexane. The extract is then processed by the same procedure used for water extracts [5].

## Discussion

The methodologies described above are straightforward, but a unique set of problems emerge when attempting to quantitate TBT at low ng L^−1^ concentrations. For instance, most commercial supplies of the Grignard reagent contain organotins as contaminants. This occurs because the same inert atmosphere apparatus is often used to synthesize organotins and Grignard reagents. Some commercial Grignard reagents were found to contain TBT at levels as high as several mg kg^−1^. A contamination-free commercial source of n-hexyl magnesium bromide has since been found which now allows us to quantify TBT in water at less than 1 ng L^−1^.

Sediment and tissue analyses are more complex than those for water. The material is difficult to extract from these matrices and the analyst must be cautious not to degrade or alter the substance in the process. Additionally, compounds that may interfere with TBT analyses are more abundant in tissue and sediment than in water. This is particularly true for samples collected from marinas and harbors which experience hydrocarbon pollution from oil spills and bilge waters.

**Figure 1:**
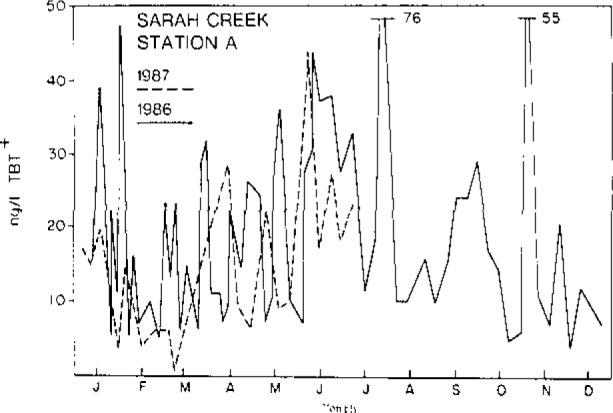
Tributyltin in near surface waters from Sarah Creek, VA over an 18-month period.

Sampling frequency is an important aspect of any analytical investigation involving chemical contamination of the natural environment. Our work with TBT confirms this statement. The natural variability of TBT concentrations in water, sediment or tissue is large. Table 1 shows the concentrations of TBT in 19 individual oysters, *Crassostrea virginica*, collected from a single location in the James River, VA [6]. The lowest concentration differs from the highest by more than a factor of two. Water samples collected at the same location and at the same phase of the tide can vary by an order of magnitude within a one week period. Samples collected at the same time but a few hundred meters apart can be vastly different in concentration [7]. There are a number of factors responsible for this natural variability. These include wind conditions which influence both the amplitude of the tides and the mixing of the water mass, the ever-changing number of vessels in the vicinity of the sampling sites and the age of the paint films, since the release of TBT from the paints is somewhat time dependent. Figure 1 shows the concentration of TBT in surface water (30 cm below surface) collected from the same location on high slack tides over an 18-month period. Such data indicate that TBT water monitoring programs, and perhaps monitoring programs for other dissolved constituents, must be carefully designed with particular attention given to natural variability if short-term trend analyses (e.g., over 1 or 2 years) are desired.

The proper interpretation of environmental chemical monitoring data depends not only on the accuracy and precision of the analyses but also on how well the samples represent the system being investigated. The authors suggest that more attention should be given to sampling in the total analytical scheme.

## Figures and Tables

**Table 1:** Concentrations of tributyltin in oysters, *Crassostrea virginica*, from a single location in the James River, VA

Oyster	Tributyltin concentration^a^ µg kg^−1^
1	301
2	285
3	211
4	515
5	280
6	345
7	386
8	254
9	374
10	258
11	411
12	446
13	480
14	311
15	264
16	379
17	373
18	325
19	325
	$\bar{x}\pm \text{sd}=343\pm 81$

a Concentrations given as TBT, dry weight.

## References

[1] Unger MA, MacIntyre WG, Huggett RJ (1987). Equilibrium Sorption of Tributyltin Chloride by Chesapeake Bay Sediments.

[2] Maguire RJ, Chau Y, Bengert GA, Hale EJ, Wong PTS, Kramer O (1982). Environ Sci Tech.

[3] Matthias CL, Olson GJ, Brinckman FE, Bellama JM (1985). A Comprehensive Method for the Determination of Aquatic Butyltin and Butylmethyltin Species at Ultra-Trace Levels Using Simultaneous Hybridization/Extraction with G C/FPD.

[4] Unger MA, MacIntyre WG, Greaves J, Huggett RJ (1986). Chemosphere.

[5] Rice CD, Espourteilie FA, Huggett RJ Appl Organometallic Chem.

[6] Espourteilie FA Tributyltin in Oysters, Crassostrea virginica,and Sediments from the Chesapeake Bay, Masters Thesis.

[7] Huggett RJ, Unger MA, Westbrook DJ (1986). Organotin Concentrations in the Southern Chesapeake Bay.

